# Gastro-Radiography—Metallic Meals

**Published:** 1914-01-17

**Authors:** 


### "MODERN METHODS" SERIES.

### Gastro-Radiography—Metallic Meals.

The correct understanding of what is seen when the stomach is examined through the *x*-ray screen after a bismuth meal is far from easy, and, for practical purposes, outside the reach of those who are not devoting special attention to the matter. Even the experts have not uncommonly differed amongst themselves as to the meaning of the shadows seen in an abdominal skiagraph at different periods after the digestion of bismuth, and considering that surgeons have come to depend so much on the results of these examinations, the responsibility thrown on the radiologists lately has become increasingly great; so much so, that they have been compelled to seek some means of coming to an agreement as to the technique of the bismuth method, as well as in regard to the common interpretation of gastric skiagrams. As an important step in this direction those who have become most closely associated with *x*-ray work in this country have come to the conclusion that some standardisation of the meal itself is essential to a proper basis for the explanation of results.

Hitherto, individual radiologists have followed their own inclinations in the matter; so that whilst some have given two ounces of bismuth mixture, others have given up to four or five ounces as a preliminary dose, the vehicle used for its administration having been variously porridge, bread and milk, jelly or sugary emulsions. Moreover, on the Continent gum in solution has been made use of to hold the bismuth in suspension, and some American specialists have relied on buttermilk for the same purpose.

### Special Conferences.

Consequently, the *x*-ray experts in this country have lately been meeting to discuss this matter, impetus having been given to their investigations by the suggestions of Dr. A. H. Hertz at last summer's British Medical Association Congress, when he pointed out the difficulties likely to arise from haphazard use of all sorts of bismuth mixtures. The development of the method obviously owes so much to Hertz's work at Guy's Hospital and elsewhere that his opinions on the point at issue have naturally carried exceptional weight.

The Royal Society of Medicine (electro-therapeutic section) has also been deliberating on the matter, and, although no general standard for the metallic meal has been agreed upon, has appointed a special committee to report on it. One of the points to which attention has been drawn is that in dealing with heavy metallic salts there is every possibility that artificial distortion of the stomach may be produced by the weight of the meal given. Consequently, it has to be decided which of the lighter salts of bismuth or barium, and the smallest doses of the same, can be of most practical use.

Of course, sufficient bismuth has to be given to form a coating to the stomach and intestines thick enough to reveal the outline of these organs under *x*-ray examination up to so long as thirty-six hours after it has been swallowed An interesting point upon which there appears to have been general agreement at all the discussions that have taken place is the necessity for administering a potion that shall not be unpleasant to the patient, because it has been found that a nauseous mixture will so upset the latter that the stomach may behave abnormally for some little time. Roughly speaking most radiologists present their bismuth as a mixture made up in half or three parts of a tumbler of porridge or bread and milk, little difficulty being found in getting the average patient to swallow the whole with a fairly good grace. Clearly enough, some slight variation will have to be permitted, and, indeed, will not affect the ultimate result, but there can be little doubt that very soon it will be out of the question for any medical man who wishes to make a scientific report on the condition of the stomach and intestines to give haphazard mixtures of bismuth, or any other metal, for the purposes of an *x*-ray examination.

### Puerperal Sepsis.

The general practitioner is likely to be assisted in the immediate future in regard to that bane of his existence puerperal sepsis by certain bacteriological methods now being worked out at the London Hospital. These tend in two important directions, firstly, to the raising of the lying-in patient's resistance to streptococcic invasion; and, secondly, the combating of puerperal infections that have unfortunately become established. A number of patients have been injected with vaccines prepared so as to increase their resistance to special strains of streptococci, but the results of the method will obviously not be easy to judge, as there must inevitably be some difficulty in knowing whether any supposed successful cases have been seriously exposed to infection or not through abnormal tearing of tissues, or opening up of blood vessels and lymphatics. A very large series of cases must, therefore, be thus treated, and the observations extended over a considerable time, before the possibilities of this prophylactic measure can be accurately estimated.

### Continuous Counter-Irritation.

The treatment of rheumatoid arthritis is admittedly unsatisfactory at the present time, although fairly successful results are obtained in certain cases by one or other of the various measures in use. It does not appear, however, that the use of continuous counter-irritation has been given a sufficiently wide trial in this country, considering the remarkable results that some practitioners are obtaining with it. Thus, cases shown recently at the Royal Society of Medicine by Dr. W. J. Midelton, who has given considerable attention to the subject, demonstrated clearly enough how on occasion patients suffering from very severe polyarthritis will recover the use of their limbs, and take up the ordinary activities of life, after treatment by this method.

